# Why don’t we provide the best treatment for chronic kidney disease?

**DOI:** 10.1590/2175-8239-JBN-2026-E014en

**Published:** 2026-08-10

**Authors:** Gisele Meinerz, Valter Duro Garcia

**Affiliations:** 1 Santa Casa de Porto Alegre, Porto Alegre, RS, Brazil.

An efficient kidney transplant (KT) program within a region or country is based on three essential pillars: donor procurement (availability of organs for transplantation), waitlist enrollment (patients eligible for transplantation), and transplant procedures (utilization of available organs). Even though KT is recognized as the kidney replacement therapy with better outcomes, better quality of life, and lower cost^1^, access to KT remains limited, and the annual number of donors and procedures performed does not match the growing demand for organs^2^.

The exact number of patients with chronic kidney disease undergoing dialysis who require KT and should be waitlisted in Brazil is not known. Two studies have addressed this issue: one conducted in the state of Rio Grande do Sul in 1990^3^, demonstrating that KT was indicated for 35% of prevalent dialysis patients and 55% of incident patients; and another carried out in Rondônia in 2015^4^, showing that 43% of the 620 patients receiving dialysis in the state were eligible for transplantation. Based on these findings, it may be estimated that approximately 40% of prevalent dialysis patients should be on the KT waiting list, while 55% of incident patients should be enrolled each year.

According to the Brazilian Annual Dialysis Census published by the Brazilian Society of Nephrology (SBN) (https://www.censo-sbn.org.br/censosAnteriores), there were 173,408 prevalent and 46,608 incident dialysis patients in 2025. In the same year, the Brazilian Transplant Registry of the Brazilian Association of Organ Transplantation (ABTO)^2^ reported 38,925 patients on the KT waiting list, corresponding to only 22% of patients undergoing dialysis nationwide—approximately half of the estimated number of patients that should have been listed. A total of 16,231 patients were added to the waiting list in 2025, slightly more than half of the projected annual number of enrollments.

Santos et al.^5^ investigated the reasons for non-enrollment on the waiting list from the patients’ perspective and identified lack of information and difficulties in undergoing the required evaluations as the main barriers. Complementing this view, Nogueira et al.^6^ provide the perspective of nephrologists, who serve as the starting point for identifying and referring patients to transplant centers. Their survey, conducted with nephrologists practicing in the state of Minas Gerais through a voluntary questionnaire, achieved a response rate of 16%. Among the reasons reported for non-referral, most respondents (90.8%) cited contraindications to KT or a perception that the risks outweighed the benefits (43.4%). Of concern, the highest proportion of unexpected responses was observed in questions related to contraindications to KT, with one-third of participants failing to correctly identify half of the contraindications.

The dialysis population is becoming older^7^, with a high prevalence of diabetes and hypertension, and the perception that remaining on dialysis may represent a lower risk than KT warrants careful evaluation by specialized multidisciplinary teams. More than 80% of respondents stated that they routinely offer KT as a renal replacement therapy modality; however, one-quarter were unable to report the actual proportion of their patients who were effectively waitlisted.

In a country as large and regionally diverse as Brazil, public health policies must be grounded in reliable data, aiming for equity and fairness in access to treatment for all patients. Minas Gerais ranks among the Brazilian states with the highest number of kidney transplants performed^2^, and the profile described by Nogueira et al.^6^ reflects professionals with solid nephrology training and extensive experience in advanced chronic kidney disease care. It should also be noted that participation in the survey was voluntary, potentially introducing response bias toward individuals with a greater interest in the subject. If these experienced professionals identified contraindications to KT as the main barrier to referral, and one-third failed to correctly answer half of the questions in this domain, this should raise concern.

Amid discussions about scarcity of dialysis slots and insufficient public funding, and with KT recognized as a more cost-effective treatment with better outcomes, studies such as that of Nogueira et al.^6^ are more necessary than ever. Understanding our reality is essential for identifying strategies to improve access to KT. Considerable emphasis has been placed on family refusal of organ donation as a primary target for public education in order to improve transplantation rates in Brazil. However, transplantation can only occur if patients are properly identified, referred, evaluated, and ultimately waitlisted. In this context, continuous multiprofessional education on KT, along with closer integration between dialysis units and transplant centers to align referral pathways and facilitate preliminary case discussions, may help reduce barriers to appropriate referral.

## Data Availability

No new data were generated or analyzed in this study.
