# Supplementary material for: Why don’t we provide the best treatment for chronic kidney disease?
Source: J Bras Nefrol. 2026 Aug 10;48(3):e2026E014. doi: 10.1590/2175-8239-JBN-2026-E014en (PMC13459822; doi:10.1590/2175-8239-JBN-2026-E014en)
Supplement: Translation

# Translation: Por que não oferecemos o tratamento ideal para a doença renal crônica?

**DOI:** 10.1590/2175-8239-JBN-2026-E014pt

**Authors:** Gisele Meinerz, Valter Duro Garcia

**Affiliations:** 1 Santa Casa de Porto Alegre, Porto Alegre, RS, Brasil.

Um programa eficiente de transplante renal (TR) em uma região ou país baseia-se no tripé: busca de doadores (órgãos para transplante), ingresso em lista de espera (pacientes para transplante) e realização dos transplantes (aproveitamento dos órgãos disponíveis). Mesmo sendo reconhecido como a modalidade de terapia renal substitutiva com melhores resultados, melhor qualidade de vida e menor custo^1^, o acesso ao TR ainda é limitado, e o número de doadores e de procedimentos realizados anualmente não consegue acompanhar a crescente demanda por órgãos^2^.

O número exato de pacientes renais crônicos em diálise que necessitam de TR e deveriam estar em lista de espera no Brasil não é conhecido. Há dois estudos sobre o assunto, um no RS, em 1990^3^, demonstrando que o TR estava indicado para 35% dos pacientes prevalentes e para 55% dos incidentes em diálise, e outro em Rondônia, em 2015^4^, que verificou que 43% dos 620 pacientes em diálise no estado tinham indicação para o procedimento. Dessa forma, pode-se estimar que cerca de 40% dos pacientes prevalentes em diálise deveriam estar em lista de espera e que 55% dos incidentes em diálise deveriam ingressar nessa lista anualmente.

De acordo com o Censo Anual Brasileiro de Diálise da SBN (https://www.censo-sbn.org.br/censosAnteriores), em 2025 havia 173.408 pacientes prevalentes e 46.608 incidentes em diálise. No mesmo ano, o Registro Brasileiro de Transplantes da ABTO^2^ reportou 38.925 pacientes em lista de espera para TR, correspondendo a 22% dos indivíduos em programa de diálise no país, metade do número estimado de pacientes que deveriam estar em lista de espera. Foram registrados 16.231 ingressos em lista em 2025, pouco mais da metade do estimado como ingresso anual.

Santos et al.^5^ levantaram as causas de não ingresso em lista pela perspectiva dos pacientes e encontraram desinformação e dificuldade de acesso aos exames como os principais entraves. Complementando esta visão, Nogueira et al.^6^ nos trazem a perspectiva do nefrologista, que representa o primeiro passo na identificação e no encaminhamento dos pacientes aos centros de transplante. O levantamento foi realizado com nefrologistas atuantes no estado de Minas Gerais, por meio de questionário de resposta voluntária, com uma taxa de resposta de 16%. Dentre os motivos para o não encaminhamento, a maioria das respostas (90,8%) foi por considerar que haveria contraindicação ao TR ou por considerar o risco maior que o benefício (43,4%). Preocupa o fato de que, justamente nas perguntas relativas às contraindicações ao TR, foi encontrado o maior percentual de respostas inesperadas, com um terço dos participantes não identificando corretamente metade das contraindicações.

A população em diálise tem se tornado cada vez mais idosa^7^ e com elevada prevalência de diabetes e hipertensão, e a percepção de que há maior risco do que benefício em permanecer em diálise, em relação ao TR, merece ser avaliada com cautela por uma equipe multidisciplinar e especializada. Mais de 80% dos respondentes afirmaram oferecer o TR rotineiramente como modalidade de TRS, mas um quarto não soube informar qual era o percentual de pacientes efetivamente inscritos em lista de espera.

Em um país com as dimensões e disparidades regionais que o Brasil apresenta, as políticas públicas de saúde devem ser embasadas em dados confiáveis, buscando a equidade e a justiça no acesso aos tratamentos para todos os pacientes. Minas Gerais figura entre os estados com maior número de TRs no Brasil^2^, e o perfil demonstrado por Nogueira et al.^6^ é de profissionais com boa formação em nefrologia e ampla atuação na DRC avançada. Vale lembrar que a amostragem foi por adesão ao questionário, o que pode trazer viés de resposta, com participantes que têm interesse no assunto. Se estes profissionais experientes responderam que a principal barreira ao encaminhamento para TR é a presença de contraindicação ao procedimento, e um terço não respondeu corretamente a metade das perguntas sobre esse tema, um alerta se acende.

Se falamos em escassez de vagas de diálise e em financiamento público deficitário, e temos o TR como uma modalidade de tratamento mais custo-efetiva e com melhores resultados, levantamentos como o de Nogueira et al.^6^ tornam-se cada vez mais necessários. Conhecer a nossa realidade é primordial para podermos identificar estratégias que visem melhorar o acesso ao TR. Focamos na recusa dos familiares à doação de órgãos como um dos principais alvos das ações de educação da sociedade para mudar o panorama dos transplantes no Brasil, mas, para transplantar, o paciente precisa ser identificado, encaminhado, avaliado e efetivamente listado. Dessa forma, a educação continuada multiprofissional sobre TR e a aproximação entre centros de diálise e centros de transplante, para alinhamento de fluxos e avaliação preliminar/discussão de casos de potenciais pacientes, podem ajudar a reduzir as barreiras ao encaminhamento adequado.

## Data Availability

Nenhum dado novo foi gerado ou analisado neste estudo.
